# The evolving role of endoscopic ultrasound from diagnosis to therapy in hepatic vascular interventions

**DOI:** 10.3389/fmed.2026.1742411

**Published:** 2026-02-24

**Authors:** Shixue Xu, Jitong Jiang, Jintao Guo, Sheng Wang, Nan Ge, Rongmin Xu, Fan Yang

**Affiliations:** Department of Gastroenterology, Shengjing Hospital of China Medical University, Shenyang, Liaoning, China

**Keywords:** endohepatology, endoscopic ultrasound, liver cirrhosis, portal hypertension, vascular intervention

## Abstract

Endohepatology, the integration of advanced endoscopy into hepatology, is rapidly evolving beyond standard diagnostics. This review focuses on its burgeoning vascular applications, particularly through endoscopic ultrasound (EUS). EUS-guided portal pressure gradient measurement (EUS-PPG) is establishing a new paradigm for direct portal hypertension assessment. Therapeutically, EUS enables precise vascular interventions such as coil and glue embolization for gastric varices and spontaneous portosystemic shunts, moving beyond blind techniques. Pioneering, bench-to-bedside procedures like the creation of EUS-guided intrahepatic portosystemic shunts (EUS-IPS) represent the next frontier. While technical complexity remains, EUS is a transformative platform within endohepatology. This article explores these cutting-edge applications and prognosticates future techniques set to redefine the management of complex hepatobiliary and vascular diseases.

## Introduction

The rapid evolution of endoscopic ultrasound (EUS) is fundamentally reshaping the landscape of hepatology management (1). By leveraging its high-resolution imaging capabilities and real-time guidance, EUS provides detailed visualization of the hepatic parenchyma, vascular structures, and collateral circulation, while enabling precise targeting from within the gastrointestinal lumen (2). This technological advantage has established EUS as a unique platform for precise and minimally invasive hepatic interventions. Its applications have expanded beyond initial diagnostic roles - such as EUS-guided liver biopsy (3, 4) and liver stiffness measurement (5–7)—into therapeutic domains, thereby introducing novel paradigms for intervention in liver diseases.

As a major cause of morbidity and mortality in patients with chronic liver disease, portal hypertension involves complex pathophysiology, including increased intrahepatic resistance, elevated portal venous inflow, and the development of portosystemic collaterals (8). Accurate assessment of portal pressure and effective management of complications—such as variceal hemorrhage, ascites, and hepatic encephalopathy—are critical for improving patient outcomes (9). However, conventional approaches face significant limitations: hepatic venous pressure gradient (HVPG) measurement is invasive, endoscopic therapies are often blind, and techniques such as transjugular intrahepatic portosystemic shunt (TIPS) require substantial expertise and involve radiation exposure (9, 10).

EUS-guided vascular interventions have emerged as a promising solution to these challenges. The field has progressed from purely diagnostic applications, such as portal pressure gradient (PPG) measurement and blood sampling, to a comprehensive therapeutic platform capable of performing variceal embolization, spontaneous shunt occlusion, and even experimental creation of intrahepatic portosystemic shunts (11). This review systematically examines the evolving role of EUS in vascular interventions, with a focus on its technical advances, clinical efficacy, and future directions in the management of portal hypertension and related complications, offering a comprehensive perspective on the integration of this cutting-edge technology into hepatology practice.

## EUS-guided diagnostic applications

### EUS-portal pressure gradient (PPG) measurement

In patients with advanced chronic liver disease, the progression to clinically significant portal hypertension (CSPH), characterized by hepatic venous pressure gradient (HVPG) ≥ 10 mmHg, poses a significant risk for decompensation and mortality (12, 13). The accurate measurement of portal pressure is crucial for the early diagnosis of portal hypertension. The gold standard for portal pressure assessment remains HVPG measurement, a technique with well-validated prognostic value and standardized operational protocols from international guidelines (12). EUS-PPG as a direct measurement method has been developed to address the invasiveness and technical requirements of HVPG, with evidence evolving from early experimental studies to multicenter clinical observations. In preclinical and first-in-human studies, Huang et al. (14, 15) pioneered a novel EUS-guided system for direct PPG measurement using a 25-gauge FNA needle and a compact manometer, establishing its initial feasibility. Furthermore, the feasibility of using a 22-gauge needle for this procedure was subsequently confirmed (16). A growing body of evidence demonstrates that EUS-PPG is a safe and reliable method for direct portal pressure gradient measurement in patients with cirrhosis, representing a valid alternative to HVPG (16, 17). Choi et al. (18) and Luo et al. (19) further reported the excellent correlation of EUS-PPG with disease severity across histological, clinical, and imaging parameters, alongside a favorable safety record. Recently, a multicenter study enrolled 373 patients confirmed that EUS-PPG measurement is technically feasible, safe, and demonstrates strong correlations with clinical parameters of portal hypertension and liver histology. Notably, an EUS-PPG > 5 mmHg was the strongest predictor of cirrhosis, outperforming established non-invasive fibrosis markers (20). These reports indicate that the technique is safe and reliable for direct portal pressure gradient measurement in cirrhotic patients and can serve as a potential complementary alternative to HVPG in selected clinical scenarios, though it has not been endorsed by Baveno VII (12) or AASLD 2024 (9) guidelines for routine use (21).

Despite these promising findings, the current evidence base for EUS-PPG has notable limitations that prevent it from replacing HVPG in routine clinical practice. First, all available studies are retrospective in design, with no prospective randomized controlled trials comparing EUS-PPG and HVPG in terms of long-term prognostic value. Second, there is a lack of standardized operational protocols for EUS-PPG: needle selection (22G vs. 25G), puncture site (portal vein branch selection), and manometer calibration methods vary across studies, leading to potential heterogeneity in measurement results. Third, selection bias cannot be excluded in existing cohorts, as most studies were conducted in tertiary referral centers with experienced interventional endoscopists, and the feasibility and accuracy in low-volume centers remain untested. Fourth, the correlation between EUS-PPG and HVPG in specific subgroups (e.g., patients with portal vein thrombosis, cavernous transformation, or acute decompensation) has not been fully elucidated.

### EUS-portal venous blood sampling

Beyond pressure measurement, EUS-guided portal vein access enables blood sampling for emerging applications such as circulating tumor cell (CTC) analysis. This approach holds promise for refining treatment strategies in intractable cancers like pancreatic and biliary malignancies, where imaging has limited sensitivity for detecting minute liver metastases. Preliminary studies have established the safety and feasibility of this technique. Catenacci et al. (22) demonstrated that EUS-guided portal venous blood sampling is safe and yields a higher CTC count than peripheral blood in pancreatic cancer patients. Further building on this, Zhang et al. (23) confirmed that not only are portal vein CTCs more frequently detected in pancreatic cancer patients compared to those with benign disease, but their numbers also correlate positively with lymph node metastasis, distant metastasis, and advanced TNM stage. While these findings underscore the significant diagnostic and prognostic potential of portal vein CTCs, their clinical significance remains investigational. A positive finding does not currently equate to imminent radiologic metastasis or alter formal staging. Future large-scale cohort studies are warranted to validate these findings and define their clinical utility. Parallel developments in EUS-guided fine-needle aspiration of portal vein thrombosis for cancer staging also highlight the expanding diagnostic potential of this approach (24, 25).

## EUS-guided therapeutic interventions for portal hypertension

Beyond its diagnostic capabilities, EUS has emerged as a powerful therapeutic tool for the management of variceal hemorrhage, offering a paradigm shift from blind endoscopic techniques to targeted, image-guided interventions (26).

### Esophageal varices

EUS-guided sclerotherapy (EUS-ES) for esophageal varices (EV) was first reported in 2000 (27) and subsequently shown in a randomized trial to obliterate para-esophageal perforating veins and reduce recurrence (28). EUS-ES yields comparable safety and efficacy to endoscopic sclerotherapy for the eradication of varices. Furthermore, it demonstrates superior long-term outcomes, characterized by a lower frequency and later onset of recurrence. The persistence of esophageal collateral vessels after sclerotherapy constitutes a key risk factor for disease recurrence (28). Nevertheless, its diffusion into routine practice remains modest. The cervical/thoracic esophageal wall averages only 2 mm in thickness, providing a narrow safety window that readily permits transmural perforation or mediastinal extravasation of sclerosant. Variceal columns are tightly packed and diminutive, mandating multiple punctures under real-time color-Doppler guidance, skills that are considerably more demanding than standard endoscopy. Procedure-related costs are triple to quadruple those of band ligation because of dedicated 19–22 G FNA needles, repeated sessions, and extended procedural time, yet overall eradication and re-bleeding rates do not differ from those achieved with ligation (29). Consequently, current guidelines continue to endorse endoscopic variceal ligation as first-line therapy, reserving EUS-ES for cases refractory to conventional treatment or for patients in whom high-risk perforating veins have been unequivocally demonstrated (12).

### Gastric varices

Gastric varices (GV) are a severe complication of portal hypertension with complex vascular anatomy and high bleeding/recurrence risk (30). Conventional blind cyanoacrylate (CA) injection is limited by incomplete obliteration and systemic embolism risk, while balloon-occluded retrograde transvenous obliteration (B-RTO) is restricted by the presence of gastrorenal shunts. EUS-guided vascular interventions (EUS-VIs) have emerged as a targeted alternative, with evidence for different therapeutic regimens evolving at distinct levels, and clinical application currently limited to complex GV cases refractory to conventional therapy (2, 31). Additionally, for patients with acute GV bleeding refractory to conventional hemostasis, EUS-guided emergency embolization can serve as a salvage therapy to rapidly control the bleeding source (32, 33).

At the technical level, EUS-VI has developed a diversified strategy centered on “coil embolization,” combined with CA, sclerosing agents, or thrombin. In 2007, Romero-Castro et al. (34) first reported EUS-guided puncture of GV feeding vessels and CA injection, confirming its safety; in 2010, the same team further introduced coil embolization and proposed a standard that the coil diameter should be 120–150% of the EUS-measured variceal diameter, which significantly reduces the risk of coil migration (35). A subsequent 6-year single-center study by Binmoeller et al. (36) involving 152 patients showed that the technical success rate of EUS-guided coil combined with CA injection exceeded 99%, the complete embolization rate of varices reached 93% (mean follow-up of 436 days), and the recurrent bleeding rate was only 3%, with no serious ectopic embolization events. The advantage of this combined regimen lies in that the coil acts as a “physical stent” to block blood flow, reducing CA dosage and the risk of its leakage into the shunt; meanwhile, CA fills the gaps between coils to achieve complete occlusion of varices. In cirrhotic patients with a history of isolated gastric fundal varices (IGV-1), bleeding, EUS-guided coil combined with CA injection exhibits excellent cyanoacrylate embolization efficiency (2.85 ± 2.05 cm^2^/mL) as shown in Li et al.’s multicenter study (37). Furthermore, the EUS-guided coil and CA combination therapy is also applicable to diverse clinical scenarios beyond secondary prophylaxis, such as primary prophylaxis in high-risk patients, rescue therapy for acute bleeding refractory to conventional CA injection and treatment for recurrent bleeding (38).

Furthermore, recent studies have explored the application of new materials, such as HydroCoil (39) containing absorbable hydrogel, whose swelling property can further enhance the blood flow-blocking effect. O’Rourke et al. (40) reported an EUS-guided coil combined with thrombin injection regimen, achieving a 95% technical success rate and 85% variceal occlusion rate in 20 patients, while avoiding CA-induced endoscopic damage, thus providing an alternative for patients intolerant to CA.

Efficacy and safety data further confirm the clinical value of EUS-VIs. Meta-analysis by McCarty et al. (41) and a randomized comparative study (42) comparing different EUS-guided treatment regimens for GVs revealed that the efficacy of coil combined with CA therapy was significantly superior to that of CA alone and coil alone, with a lower complication rate than CA alone. A multicenter retrospective study in the United States included 106 patients with bleeding or high-risk GVs, who underwent EUS-coil therapy (43). Adjunctive glue or absorbable gelatin sponge was injected in 82% of patients, ultimately achieving 100% technical success and 88.7% clinical success. Only 14.1% of patients experienced recurrent bleeding (mean 32 days), and there was no difference in efficacy between high-volume and low-volume centers, suggesting that this technique can be gradually popularized after standardized training (43).

Compared with balloon-occluded retrograde transvenous obliteration (B-RTO), EUS-VIs can complete both diagnosis and treatment in a single procedure, avoiding the cumbersome process of multiple interventional sessions required by B-RTO. Xiao et al. (44) reported a case of GVs with a huge gastrorenal shunt successfully treated by EUS coil embolization combined with B-RTO, achieving variceal occlusion without complications, indicating that EUS-VIs can synergize with interventional radiological therapy to overcome the limitations of a single technique. In comparative studies, propensity-matched multicenter analyses (32) showed EUS-VIs non-inferior to B-RTO in GV obliteration and recurrence rates, with the advantage of not relying on gastrorenal shunt anatomy. EUS-VIs also reduce the rate of extravascular injection from 60% (conventional CA injection) to <10% via real-time imaging, lowering embolism and recurrence risk. However, it is important to note that all comparative evidence to date is from retrospective propensity-matched analyses, with no head-to-head RCTs comparing EUS-VIs with B-RTO or conventional CA injection in terms of long-term outcomes.

Nevertheless, EUS-VIs still face challenges in GV treatment. Technically, the procedure requires proficiency in both EUS imaging and vascular puncture skills, resulting in a long learning curve. Additionally, the selection of puncture needles must be based on the EUS-measured variceal diameter, otherwise, coil leakage or insufficient blood flow blockage may occur. The high cost of equipment and consumables also limits their popularization in primary hospitals, and there is currently a lack of unified operational standards and training systems (30, 45).

In conclusion, current international clinical guidelines have not yet recommended EUS-VIs as first-line therapy for GV. Instead, EUS-VIs should be positioned as a second-line/salvage therapy for complex GV cases: IGV-1, GV with large gastrorenal shunts, recurrent GV after conventional CA injection, and acute GV bleeding refractory to conventional hemostasis. Its potential upgrade to first-line therapy requires large multicenter RCTs, standardization of protocols, and cost reduction of consumables.

### Ectopic varices

EUS-VIs provide a superior alternative to conventional therapies for ectopic varices, which—despite management recommendations similar to gastric varices—present unique anatomical challenges. Standard endoscopy often fails to adequately visualize deep or extraluminal lesions, resulting in low success and high recurrence rates, while interventional radiology may be hindered by difficult-to-access feeding vessels. This advantage is clearly demonstrated in the management of parastomal varices, a known source of recurrent bleeding (46). For these patients, EUS-guided therapy presents a minimally invasive option that effectively circumvents the need for high-risk surgical revision or TIPS, the latter often being contraindicated in advanced liver disease. Supporting evidence includes a retrospective study by Todd et al. (46) of 24 patients, where EUS-guided thrombin injection ± coil placement achieved 100% technical success. Over a 26.2-month follow-up, a single procedure prevented significant rebleeding in 70.8% of cases, with no major adverse events. Furthermore, the efficacy of EUS-VIs extends to other ectopic sites. Successful outcomes have been documented in cases of refractory hepatic encephalopathy from rectal varices (47), where EUS-guided sclerotherapy reduced ammonia levels and improved symptoms, and in controlling bleeding from pancreaticojejunal varices via histoacryl-lipiodol injection, with no recurrence over 1.5 years (48). These findings collectively establish EUS as a versatile and effective first-line therapeutic option for diverse ectopic varices, particularly in patients who are poor candidates for conventional interventions.

### Spontaneous portosystemic shunts

Spontaneous portosystemic shunts (SPSSs) are a common complication of severe portal hypertension, and their association with recurrent/persistent hepatic encephalopathy has led to the need for targeted occlusion therapy (49). Interventional radiology (IR)-guided shunt embolization remains the first-line and gold standard treatment for SPSSs, with well-validated efficacy and safety from multicenter studies. EUS-guided SPSS embolization has emerged as a minimally invasive experimental alternative, currently limited to first-in-human single-center experience with no further validation in larger cohorts.

The only available evidence for EUS-guided SPSS embolization comes from a first-in-human single-center cohort study involving 7 patients with symptomatic splenorenal shunts and recurrent hepatic encephalopathy. The study used EUS-guided transgastric coil + CA embolization, achieving a technical success rate of 86% (6/7 complete shunt embolization) and clinical improvement in 71% (5/7) of patients, with no serious adverse events (e.g., bleeding, embolism, perforation) reported. This preliminary study confirmed the technical feasibility of EUS-guided SPSS embolization for SPSSs adjacent to the gastrointestinal tract, and provided a potential minimally invasive option for patients who are poor candidates for IR (e.g., unfit for contrast media, inaccessible shunts via IR routes) (50).

EUS-guided SPSS embolization is currently in the early exploratory stage, with extremely limited evidence and multiple critical limitations that prevent any clinical adoption beyond tertiary referral center research. First, the evidence base is limited to a single small first-in-human cohort (*n* = 7); there are no subsequent retrospective/prospective studies, multicenter validations, or comparative analyses with IR-guided embolization. Second, selection bias is severe: the study only included patients with splenorenal shunts adjacent to the gastric wall (easily accessible via EUS), and the feasibility and safety for other types of SPSSs (e.g., paraesophageal, mesenteric) remain completely untested. Third, long-term outcomes are unknown: the study lacked long-term follow-up, and the patency rate of shunt occlusion, risk of reformation, and long-term improvement in hepatic encephalopathy are unclear. Fourth, technical standardization is absent: there are no established protocols for puncture site selection, coil/glue dosage, or post-procedure antiplatelet/anticoagulant therapy, leading to potential procedural heterogeneity. Fifth, the procedure has a narrow safety window: SPSSs are often associated with complex collateral circulation, and EUS-guided puncture may lead to inadvertent injury to adjacent vessels or organs in inexperienced hands.

### EUS-guided intrahepatic portosystemic shunt (EUS-IPS)

Building upon its diagnostic and therapeutic roles, EUS is now pioneering one of the most ambitious frontiers in interventional endoscopy: the creation of an intrahepatic portosystemic shunt. The concept was first established in animal models. Initial proof-of-concept studies by Buscaglia et al. (51) and subsequent work by Poincloux et al. (52) in porcine models demonstrated the technical feasibility of EUS-guided shunting. The procedure typically involves the simultaneous transgastric puncture of an intrahepatic portal vein branch and a hepatic vein (or the inferior vena cava)—a “skewer” technique—followed by tract dilation and deployment of a lumen-apposing metal stent (LAMS) to establish a permanent conduit for portal decompression. Notably, these preclinical studies focused solely on whether the procedure could be performed, and did not report long-term stent patency, portal pressure reduction durability, or rates of procedure-related adverse events such as stent migration, intra-abdominal bleeding, or hepatic infarction—all critical endpoints for a vascular shunt procedure.

In the human setting, EUS has not yet been validated for the *de novo* creation of a functional intrahepatic portosystemic shunt; its only clinical application related to portosystemic shunting is as a limited adjunctive tool for conventional TIPS in a small subset of patients with anatomically challenging portal venous anatomy. A pivotal example is patients with portal vein cavernous transformation (cavernoma), where the normal portal vein architecture is lost and standard TIPS access is extremely difficult. In this highly selected population, small single-center case reports (53) have described EUS-guided portal vein localization: EUS is used to percutaneously access a patent portal vein collateral, inject contrast to delineate its lumen, and position a radiopaque coil to mark the target. This maneuver provides a critical roadmap for interventional radiologists, enabling them to perform TIPS by targeting the coil and creating a safer trajectory.

Despite these promising advances, it is crucial to emphasize that the creation of a fully EUS-guided portosystemic shunt remains highly experimental (45). Significant challenges regarding procedural standardization, long-term stent patency, and most importantly, safety, require rigorous evaluation in future clinical studies. However, one can theoretically envision its potential application in geographical areas deprived of timely access to interventional radiology or in cases with exceptionally inaccessible hepatic vasculature.

## EUS-guided therapeutic interventions for non-portal hypertension

### EUS-guided portal vein stenting

In an animal model, Park et al. (54) demonstrated the feasibility of EUS-guided portal vein stenting for the treatment of portal hypertension secondary to portal vein thrombosis or tumor embolism. Their technique involved a transgastric-transhepatic puncture to access an intrahepatic portal vein branch, followed by the advancement of a guidewire into the main portal vein. Subsequently, an uncovered metallic stent was deployed under EUS guidance to achieve transluminal stenting within the occluded segment of the main portal vein.

### EUS-guided management of pseudoaneurysms

Beyond venous interventions for portal hypertension, EUS also plays a critical role in managing arterial complications in the hepatobiliary region. Pseudoaneurysms, while uncommon, represent a potentially life-threatening complication in patients with chronic liver disease. Although transarterial embolization remains the standard of care, EUS-guided therapy has emerged as a highly effective and minimally invasive alternative (55–61).

The most established techniques include direct injection of thrombin or CA glue into the sac, often combined with coil deployment. EUS-guided thrombin injection promotes rapid, localized thrombosis and is considered particularly suitable for pseudoaneurysms with a definable neck (≥3 mm), which minimizes the risk of thrombin reflux into the parent artery. A recent series by Maharshi et al. (61) reported a 87.5% (7/8) complete occlusion rate with this technique and no adverse events. For larger or wide-necked pseudoaneurysms, a combined approach using coils followed by CA or thrombin is often employed. The coils act as a scaffold to reduce turbulent flow and stabilize the thrombus, while the adjunctive agent ensures complete obliteration. Rai et al. (58, 59) successfully treated splenic artery pseudoaneurysms in six patients using this combined method after failed interventional radiology attempts, confirming complete disappearance on follow-up imaging.

These reports underscore that EUS-guided intervention offers a safe and precise therapeutic option for visceral artery pseudoaneurysms, especially when anatomical proximity to the gut lumen provides a favorable access route.

### EUS-guided intervention for malignant liver lesions

EUS-guided intervention is a minimally invasive option for malignant liver lesions (e.g., hepatocellular carcinoma, liver metastases), primarily indicated for small focal lesions (≤3 cm) in anatomically challenging sites (e.g., caudate lobe, adjacent to major vessels/bile ducts) or patients unfit for surgery/transcatheter arterial chemoembolization (62–64). Its core advantage lies in high-resolution imaging for precise targeting, enabling access to lesions inaccessible via percutaneous approaches. Key techniques include local ablation (radiofrequency ablation, ethanol injection, irreversible electroporation), fiducial placement for stereotactic body radiation therapy (success rate >90%), and experimental approaches like portal vein embolization. Efficacy data from small studies show local tumor control rates >85% for ablation, with a favorable safety profile (procedure-related complication rate <5%), mostly minor events (local pain, transient bleeding) (63). Compared to alternatives, it avoids radiation exposure, allows simultaneous biopsy and treatment, but is limited by operator expertise and inability to treat large (>3 cm) or multifocal lesions. Future directions focus on combining ablation with systemic therapies, standardizing protocols, and developing AI-assisted targeting to expand accessibility.

## Conclusion

EUS has emerged as a transformative platform for vascular intervention, demonstrating particular value in portal hypertension management while extending its applications to tumor management and vascular malformations. The technique’s unique capacity for real-time visualization and precise targeting has enabled innovative approaches to complex clinical challenges, from variceal embolization to portal pressure gradient measurement.

Despite these advances, technical complexity, limited high-level evidence, lack of standardized protocols, and high costs remain major barriers to the widespread adoption of EUS-guided vascular interventions. Future efforts should prioritize conducting prospective multicenter RCTs to validate the efficacy and long-term safety of these techniques, establishing international operational standards to reduce inter-institutional heterogeneity, developing dedicated and low-cost consumables to improve accessibility in resource-limited regions, and training interventional endoscopists to address the long learning curve. Only through these steps can EUS-guided vascular interventions realize their full potential and be integrated into routine clinical practice for the management of complex hepatobiliary and vascular diseases.
